# Tumor–stroma ratio predicts prognosis and PD-L1 expression in hepatocellular carcinoma

**DOI:** 10.1186/s12885-023-10859-6

**Published:** 2023-05-12

**Authors:** Dong Wang, Jia Luo, YiMing Tao

**Affiliations:** 1 Department of Liver Disease Center, The Affiliated Hospital of Qingdao University, No.59 Haier Road, Qingdao, Shandong 260000 China; 2 Department of General Surgery, Xiangya Hospital, Central South University, 87 Xiang Ya Road, Changsha, Hunan 410008 China; 3 Department of Hepatobiliary Surgery, Hunan Cancer Hospital, Changsha, Hunan China

**Keywords:** Tumor stroma ratio, PD-L1, Hepatocellular carcinoma, Tumor microenvironment, Prognosis

## Abstract

**Background:**

With the in-depth research on the tumor microenvironment, the tumor stroma is considered to play a leading role in malignant tumor behavior, and PD-L1 is also related to the tumor stroma. The tumor–stroma ratio (TSR) has been regarded as a novel prognostic factor in many cancers. Our study aims to assess the TSR and PD-L1 clinical value in hepatocellular carcinoma (HCC) patients.

**Methods:**

Ninety-five patients who were diagnosed with HCC were included in our study. TSR was estimated on HCC specimen hematoxylin–eosin staining (HE) sections, and the optimal TSR cut-off value was determined by receiver operating characteristic (ROC) curves. The correlation between the TSR and clinicopathologic features was also calculated. Immunohistochemistry (IHC) staining was also carried out to analyze the PD-L1 expression level in HCCs.

**Results:**

The optimal TSR cut-off value was 0.525. The median OS of the stroma-high and stroma-low groups was 27 and 36 months, respectively. The median RFS of the stroma-high and stroma-low groups was 14.5 and 27 months, respectively. In the Cox multivariate analysis, the TSR was an independent prognostic factor for HCC overall survival (OS) and recurrence-free survival (RFS) in patients who underwent liver resection. IHC staining revealed TSR-high HCC samples with high PD-L1-positive cell expression.

**Conclusions:**

Our results suggest that the TSR can predict the prognosis of HCC patients who underwent liver resection. The TSR is related to PD-L1 expression and may be a therapeutic target that can dramatically improve HCC patients’ clinical outcomes.

## Background

Hepatocellular carcinoma (HCC) is one of the most malignant tumors and has high mortality worldwide [1]. The burden of HCC is increasing globally [2], and the number of patients with HCC in China has been increasing mainly due to hepatitis virus infection. The treatment of HCC includes hepatectomy [3, 4], liver transplantation [5], radiofrequency ablation [6], transcatheter hepatic arterial chemoembolization (TACE), targeted drugs, and immune therapy [7], among others. Hepatectomy and transplantation are regarded as curative treatments for this disease [3]. Strategies have been made to improve HCC treatments and diagnosis, but the clinical outcomes of patients with HCC remain poor, with high recurrence rates [8] and high mortality. Thus, the accuracy of HCC prediction, especially for patients who undergo hepatectomy, must be further improved to obtain a better treatment effect.

Tumour–stroma ratio (TSR), which defined as the proportion of tumour cells relative to tumour stroma cells, has been confirmed as a potential prognostic factor for solid tumours [9–11]. The clinical research had found that the TSR is a more reliable parameter which can predict colon cancer clinical outcomes [12], and also in oral tongue squamous cell carcinoma [13], breast cancer [10]. Advanced basic research has shown that stromal cells in cancer play a major role as an important modulator of tumor cell growth, pathogenesis, and progression; these cells also have the potential to influence prognosis in patients with cancer [14, 15]. The tumor stroma is an important form of the tumor microenvironment (TME), which provides supportive and permissive conditions for tumor invasion and metastasis [16, 17]. Additionally, interactions between tumor cells and stromal cells result in the production of different cytokines and enzymes that play important roles in tumor growth and progression.

The different solid tumors had different TSR, althought in the same type of solid tumor, the TSR is also different. The Tumor heterogeneity has a decisive role in the poor prognosis of cancer, especially in HCC, which has significant heterogeneity [18, 19]. Tumor heterogeneity is also associated with the TSR and TME. Tumor stromal cells include fibrocytes, T cells, tumor-associated neutrophils, macrophages can interact with tumor cells and alter immune status through a variety of inflammatory factors. Moreover, advancements in research have determined that the tumor stroma can accelerate the development of the tumor [20, 21] by many signaling pathways, such as the EMT and TGF-β signaling pathways. It has been confirmed that targeting the tumor stroma may be a research direction for future antitumor therapy [22], such as cancer-associated fibroblasts may be the therapy targeting in pancreatic cancer [23]. Current studies have confirmed that TSR plays an important role in immunotherapy, which can remodel tumor immune status and accelerate tumor progression. Peritumoral stroma inactivation of PD-L1 affects the poor prognosis of HCC and is a determinant of resistance to immunotherapy [24]. Therefore, the observation of PD-L1 expression in tumor stroma is highly unusual. However, the relationship between PD-L1 and the TSR in human HCC remains unknown.

The purpose of our research was to analyze the prognostic value of tumor–stroma ratio (TSR) grading and PD-L1 expression in HCC patients who underwent hepatectomy and to explore its relationship with other prognostic factors.

## Methods

### Patient selection and data collection

Ninety-five patients who underwent hepatectomy were included in this study from Xiangya Hospital, Central South University, China. No patients in our study underwent TACE, radiofrequency ablation (RFA), or other nonsurgical treatments. Raw clinical data were collected. All patients signed informed consent. In the process of the study, we were cautious to protect the patient’s privacy. This study was approved by the ethics committee of XiangYa Hospital, Central South University.

### TSR ratio and score

To obtain an accurate percentage of TSR, we first obtained cancer percentages and then calculated the TSR using the following formula: the cancer percentage plus the tumor stroma was 100%. For example, if the cancer percentage was 60, the TSR would be 40% [25], and we take 10% as the value interval in this process. We analyzed 5-mm hematoxylin–eosin-stained sections in all tumor samples. To obtain an accurate TSR and scoring for each patient, two researchers assessed the TSR value on all tumor slides in a blinded manner. Using the 4 × objective, we selected the most invasive part on each HE slide. The researchers evaluated the TSR score by using a 10 × objective, and four visual fields were selected for the TSR score. Finally, the average value of the four visual fields was taken as the TSR ratio. The percentage of tumors mainly ranged from 20 to 80%. The above evaluation process was completed by two investigators independently, and the final result was confirmed by the third researchers in case of disagreement between the two experts.

### Immunohistochemistry (IHC)

IHC staining of PD-L1 was performed as described previously [7]. An anti-PD-L1 polyclonal antibody (ABCAM, Cat# ab205921) diluted 1:500 was used as the primary antibody. IHC staining was analyzed and scored following full-slide digitalization with Pannoramic Scan and database-linked TMA Module software (3DHISTECH, Budapest, Hungary). The numbers of PD-L1-positive cells were counted in seven respective visual fields from tissue samples with high-power fields.

### Follow-up

All HCC patients in our study had considerable clinical survival data, including recurrence time and survival data. The endpoint of the follow-up was HCC death or December 2017. OS time is taken as the time interval from the date of the operation to death. RFS was calculated from the first operation to HCC recurrence.

### Statistical analysis

The chi-square test and Student’s *t test* were used to assess the differences in patient characteristics. Survival analyses were drawn using Kaplan–Meier methods. Cox regression was used to perform univariate and multivariate analyses in HCC patients, and hazard ratios (HRs) with 95% confidence intervals (CIs) are shown in the results [26]. Statistical analyses were performed using Prism software (GraphPad Prism Software, La Jolla, CA) and SPSS 21.0 (SPSS Company, Chicago, IL) for Windows.

## Results

### The TRS cut-off value

**Fig. 1:**
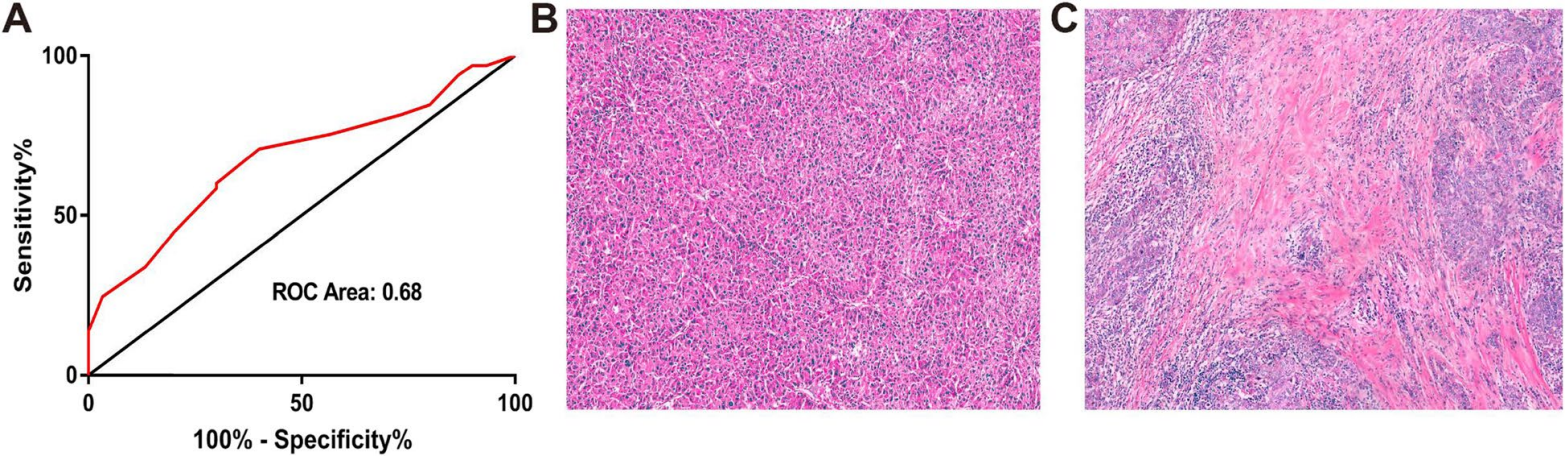
The ROC curve of the TSR and hematoxylin–eosin-stained 5-mm sections of hepatocellular carcinoma specimens. **A** The ROC curve area was 0.68 (95% CI, 0.57 to 0.79), the sensitivity was 34%, the specificity was 87%, and the optimal cutoff value was 0.525. **B** stroma-low group, **C** stroma-high group

**Fig. 2:**
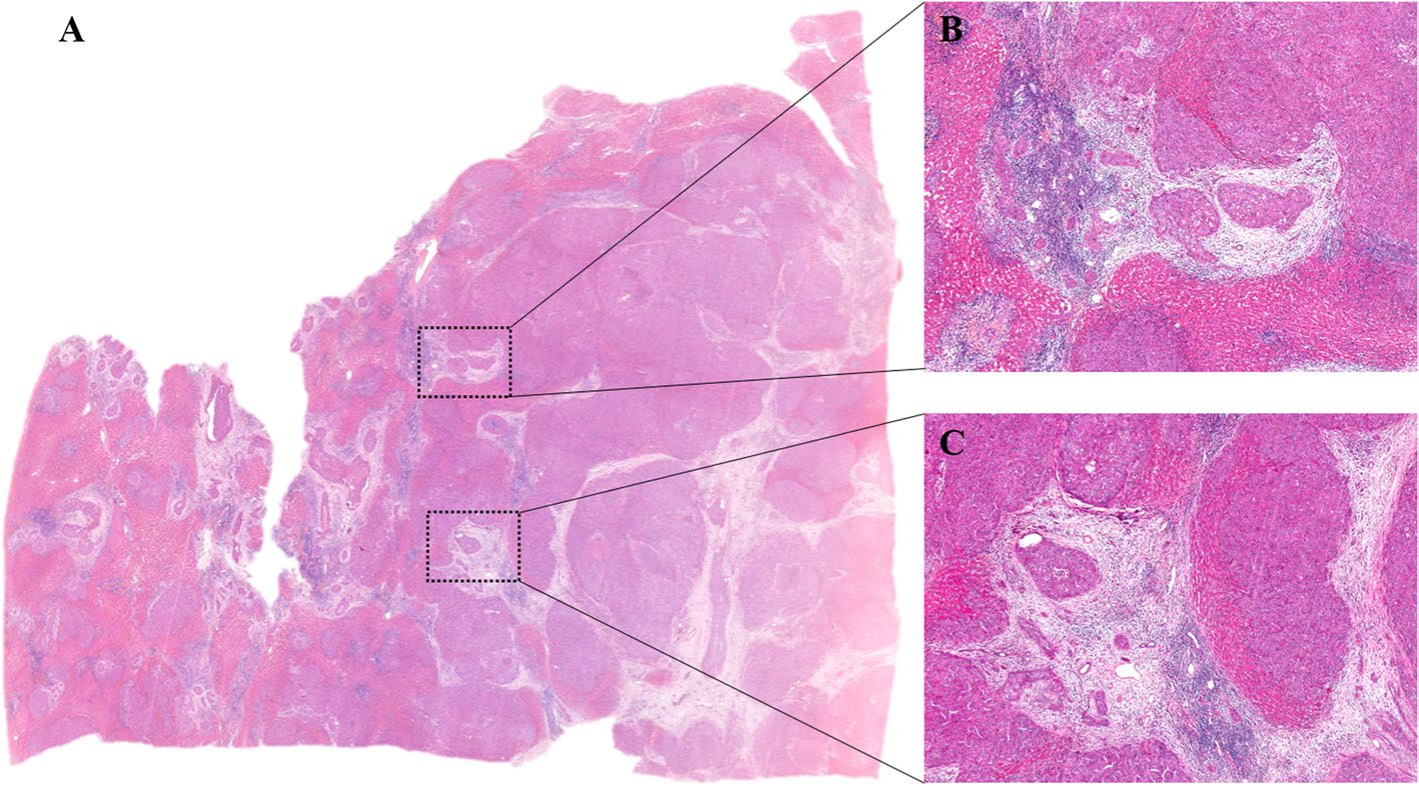
Micrometastatic nodules in HE sections. **A** shows a panoramic scan of the HE section (0.6 ×). In the section, many micrometastatic nodules are surrounded by high numbers of stroma cells. **B** and **C** show micrometastatic nodules (5 ×)

To calculate the ideal cut-off value of the TSR, we calculated the ROC curve area by OS the ROC curve area was 0.68 (95% CI, 0.57 to 0.79), the sensitivity was 34%, and the specificity was 87%. The optimal cut-off value was 0.525 (Fig. 1A). For subsequent experimental analysis, all patients were divided into the following groups: the stroma-high group (TSR > 0.525) and the stroma-low group (TSR ≤ 0.525) (Fig. 1B and C). In the stroma-high group, micrometastatic nodules were more frequent (Fig. 2). We considered that the stroma plays a leading role in HCC progression.

### The correlation of clinicopathological features and the TSR

In this study, 95 patients were enrolled, and we analyzed the clinicopathological characteristics of these patients in Table 1. The stroma-high group included 26 patients, and the stroma-low group included 69 patients. The ages of the stroma-high and stroma-low groups were 54.85 ± 9.05 years and 50.16 ± 12.31 years, respectively. The tumor sizes of the stroma-high and stroma-low groups were 5.28 ± 3.0 cm and 8.96 ± 3.69 cm, respectively. The platelet counts of the stroma-high and stroma-low groups were 167.15 ± 79.14 10^9^/L and 173.97 ± 75.68 10^9^/L, respectively. A total of 83 (86.32%) patients had CTP A stage, and 73 (74.74%) patients had single tumors. A high preoperative AFP was observed in 65 (68.42%) patients.

**Table 1:** HCC patients (*n* = 95) categorized by TSR and their clinical pathologic characteristics

**Clinical character**	**Stroma high (*n* = 26)**	**Stroma low (*n* = 69)**	***P*-value**
Age, years	54.85 ± 9.05	50.16 ± 12.31	0.08
Serum albumin, g/L	43.01 ± 6.04	441.15 ± 5.36	0.25
Tumor size, cm	5.28 ± 3.00	8.96 ± 3.69	0.00
Platelet, 10^9/L	167.15 ± 79.14	173.97 ± 75.68	0.00
ALT, U/L	48.18 ± 21.25	42.46 ± 28.96	0.36
AST, U/L	54.52 ± 20.00	48.58 ± 39.57	0.47
PT, s	13.06 ± 1.39	13.38 ± 1.16	0.32
Gender			
Male	21	59	0.39
Female	5	10	
HBsAg			
Negative	2	12	0.23
Positive	24	57	
AFP, ng/mL			
≤ 20	6	24	0.27
> 20	20	45	
CTP			
A	19	63	0.02
B	7	6	
Liver cirrhosis			
No	8	22	0.91
Yes	18	47	
Tumor encapsulation			
No	17	50	0.46
Yes	9	19	
Tumor number			
Single	13	58	0.00
Multiple	13	11	
Satellite nodules			
No	22	63	0.34
Yes	4	6	
Edmondson grade			
I–II	16	53	0.14
III–IV	10	16	
BCLC stage			
0	0	6	0.00
A	12	51	
B	9	10	
C	5	2	
TNM stage			
I	6	48	0.00
II	11	10	
III	9	11	

*HBsAg* Hepatitis B surface antigen, *AFP* α-fetoprotein, *TNM* Tumor-node-metastasis, *PT* Prothrombin time, *CTP* Child-Turcotte-Pugh, *BCLC stage* The Barcelona Clinic Liver Cancer staging, *ALT* Glutamic-pyruvic transaminase, *AST* Glutamic oxalacetic transaminase

TSR levels were closely correlated with tumor size, CTP stage, TNM stage, and BCLC stage (*P* < 0.05). There were no obvious correlations with gender, HBsAg, liver cirrhosis, serum albumin, glutamic-pyruvic transaminase (ALT), glutamic oxaloacetic transaminase (AST), etc. (*P* > 0.05) were found.

### Analysis of the RFS and OS of HCC patients who underwent hepatectomy by the TSR

**Fig. 3:**
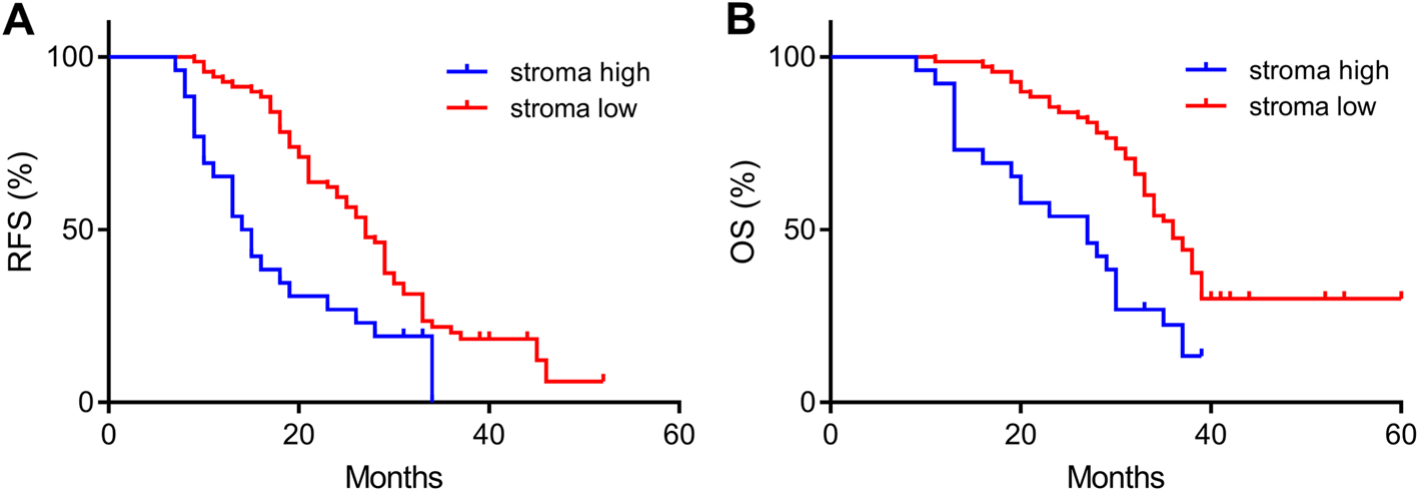
Kaplan–Meier curves of HCC patients after surgery. The median OS of the stroma-high and stroma-low groups was 27 months and 36 months, respectively (**B***P* < 0.01). The median RFS of the stroma-high and stroma-low groups was 14.5 months and 27 months, respectively (**A***P* < 0.01)

The Kaplan–Meier method was used to analyze the association between the TSR and RFS/OS (*P* < 0.01; Fig. 3). The median OS of the stroma-high and stroma-low groups was 27 months (95% CI, 0.45 to 1.25) and 36 months (0.80 to 2.23), respectively. The median RFS of the stroma-high and stroma-low groups was 14.5 months (95% CI, 0.33 to 0.88) and 27 months (95% CI, 1.14 to 3.05), respectively.

In the univariate analysis between OS and all clinicopathologic characteristics, we found that TSR (HR, 3.33, 95% CI, 0.093–0.97; *P* = 0.04), tumor size (HR, 1.33, 95% CI, 1.13–1.58; *P* < 0.001), and BCLC stage (HR, 4.93, 95% CI, 1.35–18.02; *P* = 0.02) were significant indicators for OS (Table 2). In the multivariate analysis, the TSR (HR, 8.34, 95% CI, 1.18–59.48; *P* = 0.03) was an independent prognostic factor. In the Cox multivariate analysis of RFS, the TSR was also an independent prognostic factor. The HR of the TSR was 10.06 (95% CI, 1.61–63.03; *P* = 0.02) (Table 2).

**Table 2:** Univariate and multivariate analyses of prognostic factors with RFS and OS in patients with HCC (*n* = 95)

Clinicopathologic variable	RFS		OS	
	HR (95% CI)	*P*-value	HR (95% CI)	*P*-value
**Univariate analysis**				
Gender (male vs. female)	1.38 (0.28–6.81)	0.69	2.04 (0.53–7.84)	0.30
Age, years (> 60 vs. ≤ 60)	1.02 (0.98–1.07)	0.32	1.02 (0.98–1.06)	0.40
Serum albumin, g/L (≤ 35 vs. > 35)	1.03 (0.93–1.14)	0.61	1.02 (0.94–1.10)	0.68
Platelet,10^9/L (≤ 160 vs. > 160)	0.997 (0.99–1.004)	0.12	1.001 (0.996–1.01)	0.63
ALT, U/L (≤ 50 vs. > 50)	0.98 (0.981–1.007)	0.35	0.994 (0.978–1.01)	0.44
AST, U/L (≤ 40 vs. > 40)	0.994 (0.99–1.03)	0.29	1.004 (0.99–1.017)	0.59
PT, s (≤ 13.2 vs. > 13.2)	0.64 (0.40–1.02)	0.06	0.82 (0.57–1.17)	0.27
AFP, ng/mL (> 20 vs. ≤ 20)	3.84 (0.81–18.14)	0.09	1.11 (0.44–2.85)	0.82
HBV (presence vs. absence)	2.96 (0.36–24.37)	0.31	1.78 (0.56–5.69)	0.33
TSR (> high vs. ≤ low)	1.16 (0.34–4.06)	0.82	3.33 (1.03–10.73)	0.04
BCLC stage (C vs. 0/A/B)	1.78 (0.46–6.84)	0.40	4.93 (1.35–18.02)	0.02
TNM stage (II/III vs. I)	4.75 (0.59–38.36)	0.14	3.19 (0.86–11.87)	0.08
Tumor number (multiple vs. single)	1.44 (0.44–4.65)	0.55	1.53 (0.54–4.36)	0.42
Edmondson grade (III/IV vs. I/II)	1.16 (0.34–3.98)	0.82	1.21 (0.47–3.15)	0.70
Tumor size, cm (> 5 vs. ≤ 5)	1.13 (0.95–1.34)	0.17	1.33 (1.13–1.58)	0.01
Satellite nodules (presence vs. absence)	1.27 (0.24–6.62)	0.78	4.66 (0.56–38.6)	0.15
Tumor encapsulation (none vs. complete)	1.56 (0.51–4.79)	0.44	1.04 (0.40–2.67)	0.94
Liver cirrhosis (presence vs. absence)	3.84 (0.81–18.14)	0.09	1.41 (0.54–3.67)	0.49
CTP (A vs. B)	2.69 (0.32–22.28)	0.36	6.57 (0.81–53.07)	0.08
**Multivariate analysis**				
PT, s (≤ 13.2 vs. > 13.2)	0.91 (0.76–1.10)	0.34	NA	
CTP (A vs. B)	NA		1.11 (0.52–2.37)	0.79
AFP, ng/mL (> 20 vs. ≤ 20)	1.29 (0.79–2.09)	0.31	1.13 (0.67–1.94)	0.63
Tumor size, cm (> 5 vs. ≤ 5)	1.00 (0.92–1.08)	0.35	1.05 (0.97–1.14)	0.23
TSR (> high vs. ≤ low)	10.06 (1.61–63.03)	0.02	8.34 (1.18–59.48)	0.03
BCLC (C vs. 0/A/B)	NA		2.28 (1.08–4.82)	0.31
TNM (II/III vs. I)	1.89 (0.92–3.88)	0.81	1.01 (0.44–2.32)	0.97
Liver cirrhosis (presence vs. absence)	1.12 (0.68–1.83)	0.65	1.14 (0.66–1.95)	0.63

*HBsAg* Hepatitis B surface antigen, *AFP* α-fetoprotein, *TNM* Tumor-node-metastasis, *PT* Prothrombin time, *CTP* Child-Turcotte-Pugh, *BCLC stage* The Barcelona Clinic Liver Cancer staging, *ALT* Glutamic-pyruvic transaminase, *AST* Glutamic oxalacetic transaminase

### PD-L1 in TSR-high and TSR-low HCC

**Fig. 4:**
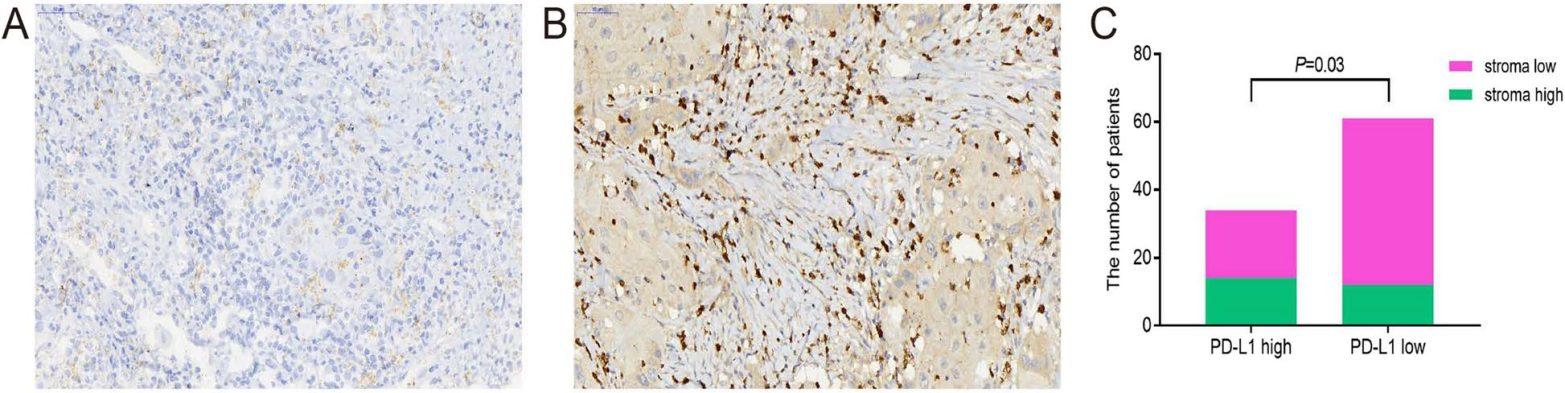
Immunohistochemical study of PD-L1. For the stroma-low group (**A** 40 × magnification) and stroma-high group (**B** 40 × magnification), chi-square analysis of the difference between the two groups (**C*** P* = 0.03)

PD-L1 is widely expressed and activated by cancer stromal cells, such as fibroblasts, T cells, B cells and macrophages. Studies have shown that PD-L1 overexpression in HCC patients is associated with tumor aggressiveness and postoperative recurrence. Does the TSR ratio affect the expression level of PDL1?In our study, we found that in the TSR high group, PD-L1 expression also increased (Fig. 4A and B). In the stroma-low group, the PD-L1 high expression rate was 28.9%, and in the stroma-high group, the PD-L1 high expression rate was 53.85%. There were significant differences between the stroma-high and stroma-low groups (*P* = 0.03, Fig. 4C).

## Discussion

In this study, we found that the TSR might be looked upon as a novel biomarker in the prediction factor of patients with HCCs after the operation. In our study, patients with a high TSR had a poor prognosis, and patients with a low TSR had good outcomes. In the TSR-high and TSR-low groups, the tumor size, tumor number, BCLC stage, and TNM stage were significantly different (*P* < 0.05), and the clinical data showed that a high TSR may be correlated with advanced-stage HCC. Additionally, the TSR in HCC may be correlated with invasiveness and metastasis. Therefore, the TSR is a significant prognostic factor for patients with HCC who undergo hepatectomy.

The tumor stroma, including cancer-associated fibroblasts, immune cells [27], epithelial cells, extracellular matrix (EMC), and extracellular molecules, can promote tumor invasion and metastasis [28]. The interactions between stromal cells and tumor cells activate various molecular signaling pathways, such as interleukin-6/STAT-3/c-Myc pathway [29], and TGF-β pathway in prostate cancer [30], lung cancer [31], and colorectal cancer [32]. And the immune cells in tumor stroma can remodel the immune microenvironment and affects tumor status and susceptibility to immunotherapy.

In China, patients with HCC mostly develop the disease from hepatitis infection, and they are often diagnosed with liver fibrosis or even cirrhosis. The severity of cirrhosis is significantly correlated with the survival time of patients with HCC after hepatectomy [26]. However, the patient number in our study was too small to reach this conclusion. We hypothesized that patients with severe cirrhosis may have a high TSR and are more likely to experience metastasis, ultimately leading to a poor prognosis. In previous studies, Marasco, G. and Xin-Fei Xu demonstrated that cirrhosis is an independent risk factor for postoperative recurrence of HCC [33, 34]. In liver cirrhosis, the proportion of stromal cells is increased. In our study, we also found that the TSR-high group had poor outcomes. Therefore, further research TSR analysis for patients with HCC, especially those with fibrosis or cirrhosis, can accurately predict HCC patient prognosis. In our research, we confirmed that the TSR is an independent risk factor for the prognosis of HCC patients who underwent hepatectomy. On the one hand, the TSR can provide more accurate prediction of patients with HCC after hepatectomy. On the other hand, we may change the postoperative management strategies for such patients who have a high TSR. For HCC patients who have a high TSR, it is possible to give adjuvant therapy after surgery, such as targeted drugs and PD-L1, and all these questions also require in-depth clinical study.

Tumor and stromal cells have mutually beneficial interactions. The growth of the tumor stroma provides the necessary support for tumor cells, while stromal cells enhance the malignant biological behavior of tumor cells, and stromal cells can also be used as therapeutic targets [22, 35]. In our research, we found that TSR-high HCC patients had poor outcomes, and we found that these patients had abundant tumor stroma, which may promote the malignant progression of HCC cells. Tumor-associated stromal fibroblasts are essential for the metastatic progression and immune surveillance escape of solid tumors, including HCC [36].

Immune checkpoint inhibitors play an important role in the treatment of HCC. However, the efficacy of immune checkpoint inhibitors varies greatly in different patients.

Which patients with HCC would benefit from immunotherapy? Current studies have found that PD-L1, TMB (tumor mutational burden), MSI (microsatellite instability), and TILs (tumor-infiltrating leukocytes) can be used as biomarkers of PD-L1 treatment [37, 38]. Whether the TSR can affect the expression of PD-L1? In our study, we found that in the TSR-high group, PD-L1 expression also increased. PD-L1 may act as a biomarker for PD-L1 treatment in HCC and play an important role in HCC therapy. We hypothesized that the stromal cells, such as cancer-associated fibroblasts, T cells, may affect the expression level of PD-L1, and may predict the immune therapy outcomes [39].

According to our study, we can predict the prognosis of HCC patients according to TSR. Patients who with high TSR may need to change the management strategy, and even underwent adjuvant therapy, such as TACE, targeted therapy. In addition, for HCC patients who with high TSR, PD-L1 therapy may improve the clinical outcomes. In the colorectal cancer, Liang Y found that TSR can predict the neoadjuvant chemoradiotherapy outcomes [40]. And the TSR can be used as biomarker for predicting the prognosis and immunotherapy in HCC patients.

In this study, in patients with a high TSR, micrometastatic nodules were more likely to be detected by microscopy. Thus, we proposed the following hypothesis: (1) the increased TSR of the TME may promote tumor metastasis because the tumor stroma provides more nutrients and growth factors necessary for migration; the stroma cells also prepare the most appropriate “soil” for tumor cells. (2) Compared with a low TSR, a high TSR may enhance malignant biological behaviors in cancer cells, and these tumors may be more prone to metastasis affected by cancer cells. (3) The high percentage of the stroma may provide greater protection for cancer cells from immune cell killing or enable cancer cells to escape the immune system and aid in therapeutic resistance. (4) The molecular signal transduction between tumor cells and stromal cells promotes the invasion ability of tumor cells. This potential mechanism may account for the poor clinical prognosis in patients with HCC who have a high TSR.

In this research, we confirmed that the TSR is an independent factor in predicting outcomes in HCC patients who underwent hepatectomy. PD-L1 expression is related to the TSR, and the tumor stroma may provide a new target for HCC treatment. Our research has the following shortcomings. First, our study was retrospective, and the sample size was small. Thus, statistical bias may exist. Second, the TSR cannot be accurately obtained, which may lead to variations among studies.

## Conclusions

This study suggests that the TSR can predict the prognosis of HCC patients who underwent liver resection and that the TSR is related to PD-L1 expression and may be a therapeutic target that can improve HCC patients’ clinical outcomes.

## Data Availability

The data used or analyzed during the current study are available from the corresponding author upon reasonable request.

## Abbreviations

TSR: Tumor–stroma ratio
HBsAg: Hepatitis B surface antigen
AFP: α-Fetoprotein
TNM: Tumor-node-metastasis
PT: Prothrombin time
CTP: Child-Turcotte-Pugh
BCLC stage: Barcelona Clinic Liver Cancer staging
ALT: Glutamic-pyruvic transaminase
AST: Glutamic oxaloacetic transaminase
